# Supplementary material for: The genome sequence of the Pale Oak Beauty, Hypomecis punctinalis (Scopoli, 1763)
Source: Wellcome Open Res. 2024 Sep 18;9:531. [Version 1] doi: 10.12688/wellcomeopenres.23061.1 (PMC12501592; doi:10.12688/wellcomeopenres.23061.1)
Supplement: Peer review history

# Reviewer report: Reviewer response for version 1

**DOI:** 10.21956/wellcomeopenres.25397.r132281

**Published:** 2025-10-06

**Authors:** Panagiotis Ioannidis

**Affiliations:** 1 Foundation for Research & Technology - Hellas, Crete, Greece

This manuscript describes the genome sequencing, assembly and annotation of the lepidopteran *Hypomecis punctinalis*.

 All methodology is the standard one used in DToL projects, and the resulting assembly looks good with virtually the entire assembly being found in a few chromosome-level scaffolds.

 My only comment has to do with reporting the genome annotation results. The community needs to understand that simply reporting the number of mRNAs, protein-coding genes etc is not enough! They should be also reporting the BUSCO score of this gene set, for example. Or some other metric of quality.

 Also, what is a "disjointed population"? Maybe the authors could explain more? Was it transferred there by transportation from Europe? Is it considered invasive? Is it causing problems in Asia?

Are sufficient details of methods and materials provided to allow replication by others?

Yes

Is the rationale for creating the dataset(s) clearly described?

Yes

Are the datasets clearly presented in a useable and accessible format?

Yes

Are the protocols appropriate and is the work technically sound?

Yes

Reviewer Expertise:

insect genomics; bioinformatics

I confirm that I have read this submission and believe that I have an appropriate level of expertise to confirm that it is of an acceptable scientific standard.

# Reviewer report: Reviewer response for version 1

**DOI:** 10.21956/wellcomeopenres.25397.r104954

**Published:** 2024-11-03

**Authors:** Bin Zhang

**Affiliations:** 1 China-Australia Joint Institute of Agricultural and Environmental Health, Qingdao Agricultural University, Shenzhen, China

Pale Oak Beauty is an important moth, which is widespread in south-eastern England. The sample for genome assembly is suitable to conduct the sequencing using Pacific Biosciences single-molecule HiFi long reads. According to the results, the quality of assembly is good and amount of annotation is in noraml range.  I believe this genome assembly would be a excellent data basic for further research on this moth.

Are sufficient details of methods and materials provided to allow replication by others?

Yes

Is the rationale for creating the dataset(s) clearly described?

Yes

Are the datasets clearly presented in a useable and accessible format?

Yes

Are the protocols appropriate and is the work technically sound?

Yes

Reviewer Expertise:

insect ecology and bioinformatics

I confirm that I have read this submission and believe that I have an appropriate level of expertise to confirm that it is of an acceptable scientific standard.
